# Genomic signatures of adaptation across a landscape of crickets following the introduction of a parasitoid fly

**DOI:** 10.1126/sciadv.aed1906

**Published:** 2026-09-09

**Authors:** Jack G. Rayner, Leeban H. Yusuf, Renjie Zhang, Shangzhe Zhang, Oscar E. Gaggiotti, Nathan W. Bailey

**Affiliations:** Centre for Biological Diversity, University of St Andrews, St Andrews, UK.

## Abstract

Novel species interactions provide an opportunity to assess the earliest stages of genetic adaptation. We combined population genomics and field selection surveys to explore the genomic and geographic landscape of adaptation in small, fragmented Hawaiian cricket populations, which are parasitized by larvae of an introduced fly that targets singing males. Multiple protective male-silencing cricket morphs have recently spread under this novel selection pressure, despite song’s important roles in mate attraction. We find evidence of sharp declines in cricket effective population sizes following the fly’s introduction and identify regions under spatially varying selection mediated by infestation risk, which are linked to an adaptive morph and other genes implicating in resisting infestation. Despite repeated bottlenecks, genetic variation is dominated by structural variant polymorphisms seemingly maintained under balancing selection. Our study illustrates pervasive consequences of abrupt changes in selection on small populations. Fly-mediated selection remains strong despite the spread of adaptive male-silencing phenotypes, which also reduce male fitness in the context of mate attraction.

## INTRODUCTION

Understanding the extent to which wild populations are capable of responding to abrupt changes in selection, how they do so, and the circumstances that follow are key aims in evolutionary biology. Human activity imposes selection on wild populations in a multitude of ways, but one of the most abrupt is via influencing species distributions (*1*). These perturbations can offer the otherwise rare opportunity to study populations’ responses to novel selection pressures, e.g., following the introduction of a new predator. In extreme cases, species introductions can foster the emergence of novel coevolutionary interactions, such as those between hosts and parasites, with profound evolutionary consequences (*2*). Studies that combine knowledge of the timing of an introduced selection pressure, geographical variation in its strength, and fitness consequences of phenotypic variation provide a powerful opportunity to investigate the conditions of genetic adaptation in its earliest stages (*3*–*5*).

Hawaiian populations of Oceanic field crickets, *Teleogryllus oceanicus*, have recently become engaged in a novel coevolutionary interaction with a deadly eavesdropping parasitoid fly, *Ormia ochracea*. These species are only known to co-occur in Hawaii, to which *O. ochracea* were recently introduced (*6*, *7*), and where *T. oceanicus* serve as their primary host. Within the past 25 years, multiple mutant wing morphs have emerged and spread through Hawaiian *T. oceanicus* populations, each of which strongly reduces the amplitude of male song. Reduced song amplitude protects males from being located by gravid female *O. ochracea* (*8*–*13*), which use cricket song as a phonotactic cue (*14*) and are remarkably accurate in pinpointing the source (*15*, *16*), upon which they deposit endoparasitic larvae that burrow into cricket hosts. The best characterized of these male-silencing wing morphs observed in *T. oceanicus*, “flatwing,” was discovered in 2003 in a population on the island of Kauai, in which it subsequently spread very rapidly, but has since been observed on two more islands (*8*, *17*) and has been cited as an example of evolutionary rescue in the wild (*18*). At least four more alternative male-silencing morphologies have now been described in Hawaiian *T. oceanicus*, and these now exist in a heterogeneous mixture of abundances and combinations across a mosaic of small, fragmented populations throughout the Hawaiian archipelago (*11*, *13*, *19*, *20*). Research into the genetic basis of adaptive responses to selection from *O. ochracea* has therefore focused on identifying variants underlying these mutant wing morphologies, repeatedly finding evidence of large-effect loci (*12*, *19*, *21*). Yet, although adaptive wing morphs have spread in populations across the Hawaiian archipelago, they vary notably in frequency and have rarely spread to fixation. This is likely due, in part, to strong fitness benefits of song in the context of attracting and courting females (*22*), suggesting the spread of mutant male-silencing wing morphs should depend on variation in fly attack rates. However, the prediction that the distribution and frequency of male-silencing phenotypes is influenced by local differences in selection from *O. ochracea* has not been tested.

**Fig. 1.**
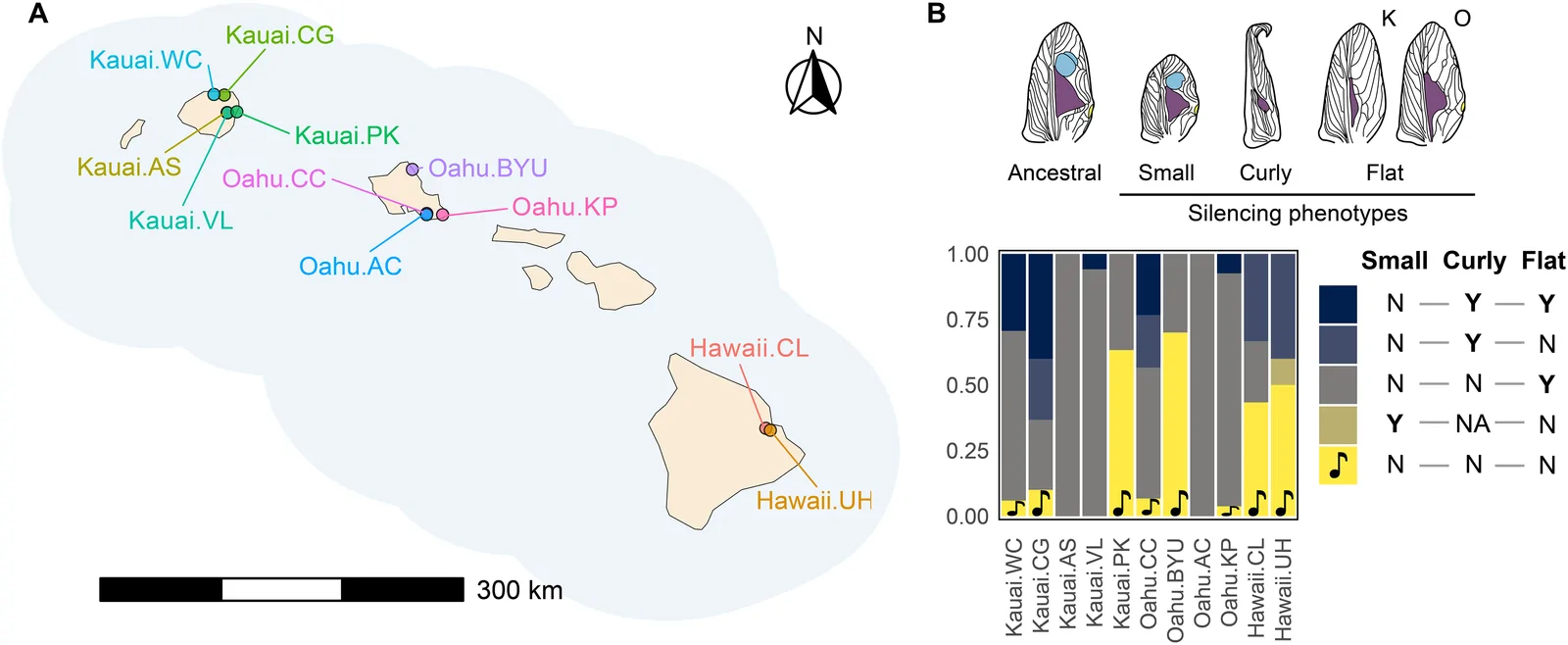
Sampling locations and morphological wing variants in Hawaiian *T. oceanicus*. (**A**) Map of Hawaii showing locations from which crickets and flies were sampled. Note that some sampling locations are nearby, but all are separated by urban or geographic barriers and multiple nearby locations show fixed differences in the presence/absence of various wing phenotypes (*19*). (**B**) Proportions of different wing phenotypes (illustrated above) represented in genome sequencing samples from each patch. Bright yellow bars represent singing-capable males, i.e., those with typical ancestral phenotypes: long wings, straight wing morphology, and normal-wing venation. Small-wing males are assigned “NA” values for wing shape because it is difficult to confidently assess. Line drawings of wing phenotypes are adapted from (*13*), with blue, purple, and yellow regions highlighting the areas of the mirror, harp, and plectrum, respectively, that influence song production. K and O labels above flatwing illustrations represent variants typically found on islands of Kauai and Oahu, respectively.

Here, we assess the genomic landscape of recent and ongoing adaptation across multiple cricket populations to evaluate support for behavioral observations indicating mutant wing morphologies spread under selection from the fly (*8*, *17*, *20*). We surveyed locations across three islands of the Hawaiian archipelago for *T. oceanicus* populations. From each location (*N* = 11; Fig. 1A), we resequenced genomes of male crickets (total *N* = 300 genomes at ∼15× depth of coverage), noting whether each expressed common male-silencing phenotypes (Fig. 1B), and recorded the number of *O. ochracea* that were attracted to cricket song playback as a measure of local selection pressure (*N* = 489 playback trials across 42 nights). We combine these data for a comprehensive study of the genomic landscape of local adaptation in wild cricket populations, illuminating the conditions that have underpinned this remarkable example of rapid and recurrent adaptation, and its evolutionary consequences.

## RESULTS

### Structural variants dominate genetic and transcriptomic variation

**Fig. 2.**
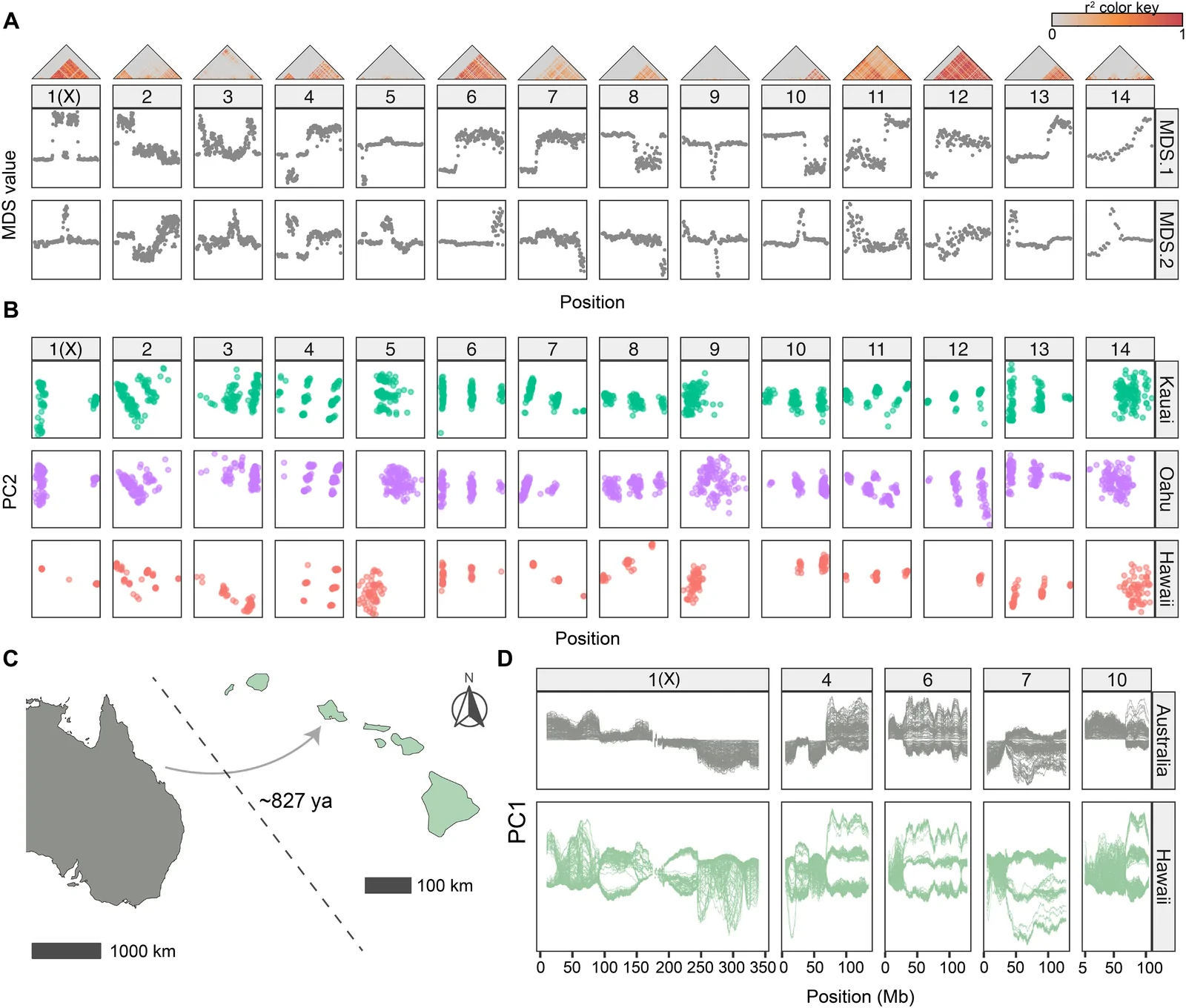
Large, polymorphic SVs in *T. oceanicus*. (**A**) Triangles show observed patterns of linkage disequilibrium across 14 chromosomes, calculated from all Hawaiian samples. Panels below show MDS values, with discrete tracts of divergence supporting evidence of large SVs (*23*). (**B**) PCA plots by chromosome, separated by island, with discrete clustering of inferred karyotypes into two or three groups showing that SVs are typically polymorphic on each island. (**C**) Map illustrating the estimated divergence of Australian and Hawaiian *T. oceanicus* populations ∼830 years ago (ya). (**D**) First principal component from windowed PCA (*82*), illustrating patterns of genomic divergence between individuals (gray lines) across a subset of chromosomes in Australian (top) and Hawaiian (bottom) *T. oceanicus*. Note that windowed PC1 values in (D) do not correspond to chromosome-wide PC1 values in (B).

Adaptive flatwing and curly-wing phenotypes have previously been found to be in linkage disequilibrium with large putative inversions on chromosome 1 (the X) and chromosome 2, respectively, though neither inversion is causally associated with the male-silencing phenotype (*19*). In initial exploratory analyses of 300 resequenced genomes, we found evidence that similar large structural variants (SVs) dominate genetic variation in Hawaiian *T. oceanicus*. These SVs were inferred on the basis of discrete regions of elevated linkage disequilibrium and heterogeneous patterns of population structure across the genome (Fig. 2A and fig. S1) (*23*), leading samples to cluster into discrete karyotypes in chromosome-wide principal components analysis (PCA) (Fig. 2B) (*24*). On the basis of these criteria, we observed at least one large (>10 Mb) SV on 11 of the 14 chromosomes. We did not observe clear differences in read depth in these regions (fig. S2), so we suspect most of these SVs to be inversions.

Hawaiian populations of *T. oceanicus* appear to have split from ancestral Australian populations just over 800 years ago (*12**,* *25*) (Fig. 2C). We investigated whether the SVs observed in Hawaii proliferated after this split or are present across the species range, incorporating previously published reduced representation sequencing (RAD sequencing) data from 170 Australian *T. oceanicus* specimens (*26**,* *27*). Windowed PCA supported evidence for both scenarios, depending on the SV. For example, polymorphic SVs on chromosomes 6, 7, 10, 12, and 13 showed evidence of being present in both regions, whereas the large inversion on chromosome 1 appears specific to Hawaii (Fig. 2D and fig. S3). SVs shared across regions showed evidence of greater genomic divergence in Hawaii, fitting expectations under elevated genetic drift in these small, isolated populations (Fig. 2D).

The size of these SVs and their persistence as polymorphisms in fragmented cricket populations across multiple Hawaiian islands, and in many cases over longer evolutionary timescales, suggests that their persistence is influenced by balancing selection, implying important effects on phenotype and fitness (*28*). We investigated evidence of pervasive phenotypic effects of these SVs using existing RNA sequencing data previously generated from neural tissue samples of 48 lab-reared crickets of both sexes (*29*). We again found evidence of large SVs dominating genetic variation on several chromosomes, and variance partitioning and differential expression analyses revealed these structural polymorphisms to have very strong local effects on gene expression (fig. S4).

### The parasitoid fly is implicated in recent cricket population declines

**Fig. 3.**
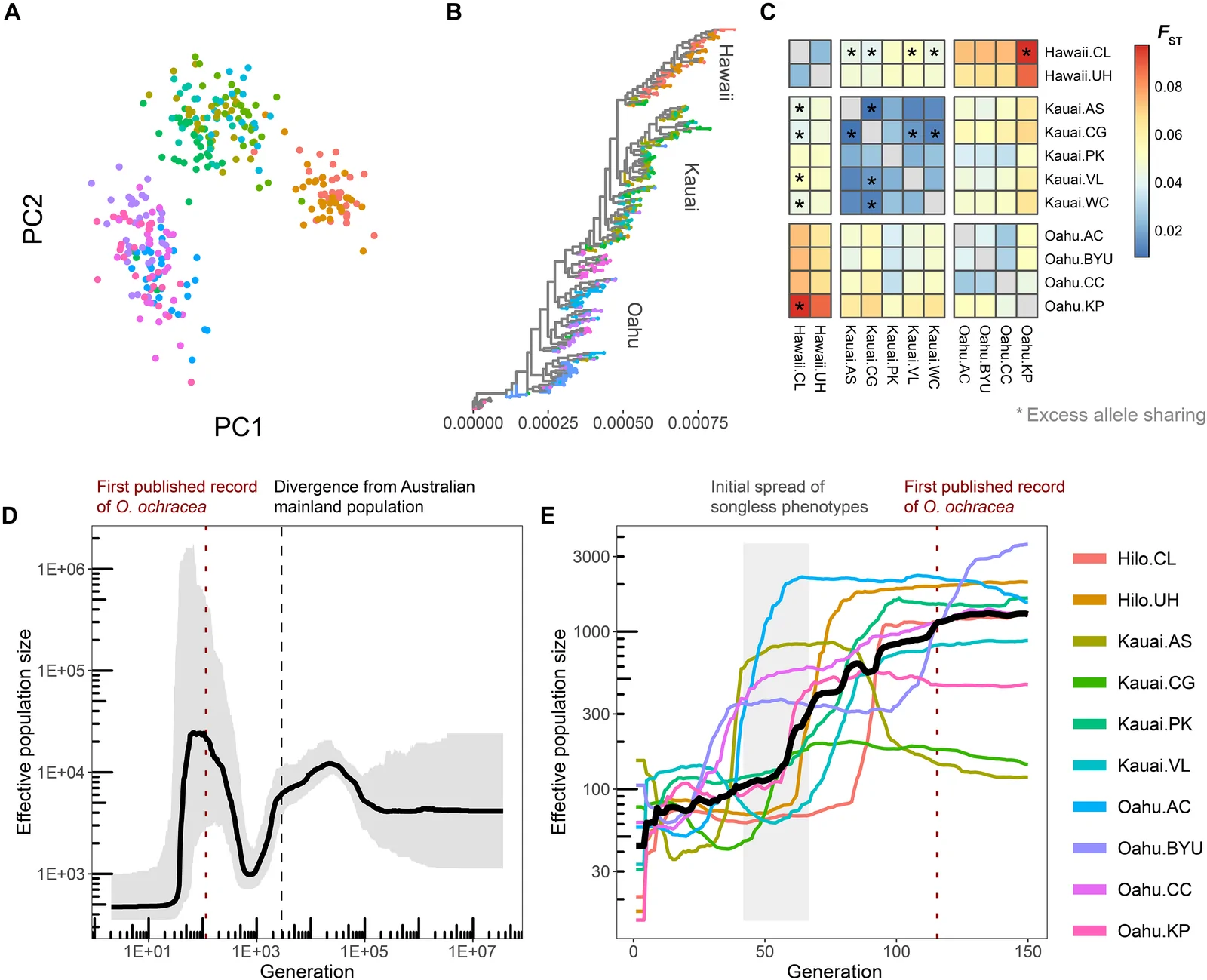
Cricket population structure and changes in effective population size. (**A**) PCA of genetic variation across 300 *T. oceanicus* males, with points colored according to sampling location. (**B**) Phylogenetic tree, rooted by the sister species *T. commodus*. (**C**) Pairwise mean *F*_ST_ values across populations. Pairs of locations with excess allele-sharing according to *F*-branch statistics are identified by asterisks (figs. S5 and S6). (**D**) Long-term changes in *N*_e_ inferred from *T. oceanicus* in Oahu.BYU. The black line shows the median estimated effective population size. The ribbon illustrates the posterior 95% confidence interval. Inferred patterns were strongly consistent in other Oahu populations (fig. S7). (**E**) Short-term changes in *N*_e_. Lines are colored according to geographic location of samples, with the black line showing the median *N*_e_ across locations.

Because patterns of genomic variation were strongly influenced by SVs, which would likely confound analyses of population structure and demographic inference, we restricted these analyses to linkage-pruned variants from chromosomes 5, 9, and 14, on which we did not observe evidence of large SVs in Hawaiian populations. PCA, approximate maximum likelihood phylogenetic trees (*30*), and pairwise *F*_ST_ analyses indicated that cricket population structure emerges predominantly at the level of the three islands: Kauai, Oahu, and Hawaii (Fig. 3). There was some evidence of finer-scale population structure between sampling locations on Oahu, on which *T. oceanicus* appear to have been first introduced (*12*). *F*-branch statistics (*31**,* *32*) supported excess allele sharing and thus evidence of gene flow between multiple locations in Kauai and between locations in Kauai and Hawaii and Oahu and Hawaii (Fig. 3C and figs. S5 and S6).

We next explored how evolutionary dynamics of Hawaiian *T. oceanicus* have been shaped by their initial colonization of the archipelago (*12**,* *25*) and by the more recent introduction of the parasitoid fly from North America; the fly was first documented in Hawaii in 1989 (*6*, *7*). Assuming an average of 3.5 cricket generations/year (*33*) indicates that Australian and Hawaiian *T. oceanicus* diverged ∼2900 generations ago, whereas the fly became prevalent in Hawaii ∼116 generations before sample collection, in 2022. Historical changes in effective population size (*N*_e_), estimated via Bayesian inference of coalescent times (*34*), supported an initial decline coinciding with the original split from Australian mainland populations, fitting expectations following genetic bottleneck and founder effects (Fig. 3D and fig. S7). Estimates of *N*_e_ before this divergence appear unexpectedly low, perhaps due to the presence of additional SVs in the core species range that affects the reliability of these estimates before divergence (fig. S3). *N*_e_ was inferred to have subsequently increased following colonization of Hawaii, until the approximate timing of the parasitoid fly’s introduction, when it fell precipitously (Fig. 3D).

The strength of declines in *N*_e_ of Hawaiian cricket populations following the fly’s recent introduction was reiterated by analyses using linkage-based inference of changes across populations (Fig. 3E), which has greater power to detect recent trends (*35*). Declines were broadly consistent across populations, and analyses of repeated random subsamples suggested inferences were robust to the individuals and variants included in the analysis (fig. S8). As well as fitting predictions following the introduction of the parasitoid fly, inferred declines correspond with recent observations of population crashes at several of these locations. In particular, we found no crickets in in our most recent surveys of Oahu.AC (June 2022) and Hawaii.CL (May 2024), each of which had previously been densely populated (*36*), and have also noted marked declines in the number of sampled crickets at Hawaii.UH and Oahu.KP. *N*_e_ declines continued during the emergence and spread of adaptive male-silencing phenotypes—which benefit male survival by evading detection from the fly—beginning ∼42 to 67 generations before sampling (Fig. 3E). Although this fits predictions, given genetic bottlenecks associated with severe selection pressures, it is also notable because, besides its negative fitness consequences via attracting parasitoid flies, male cricket song serves as an important sexual signal used by females to locate and discriminate between male mates (*19**,* *20**,* *22**,* *37*) and exerts indirect genetic effects, with higher levels of song associated with increased reproductive investment in both sexes (*38**,* *39*). Thus, declines in the frequency of singing-capable males could feasibly have exacerbated recent and ongoing declines in *N*_e_.

### Contemporary selection by parasitoid flies is variable across locations

In 489 replicate 10-min field trials across the same 11 locations, cricket song playbacks attracted 415 female flies, whereas an equal number of silent traps attracted just a single fly. Although males with mutant wing morphs such as flatwing, curly-wing and small-wing are, in at least some cases, technically capable of producing sound through stridulation, the amplitude of this “song” is reduced to near-ambient noise levels and prior research has repeatedly shown that fly traps broadcasting their song attract no more flies than those not broadcasting song (*11**,* *15**,* *40*). Our observations therefore reiterate that males expressing these mutant wing morphs are very strongly protected from parasitism, resembling findings from dissections of wild-caught Hawaiian *T. oceanicus*, in which fewer than 1% of flatwing males harbored parasitoid larvae, contrasting with >30% of singing-capable males (*8*).

**Fig. 4.**
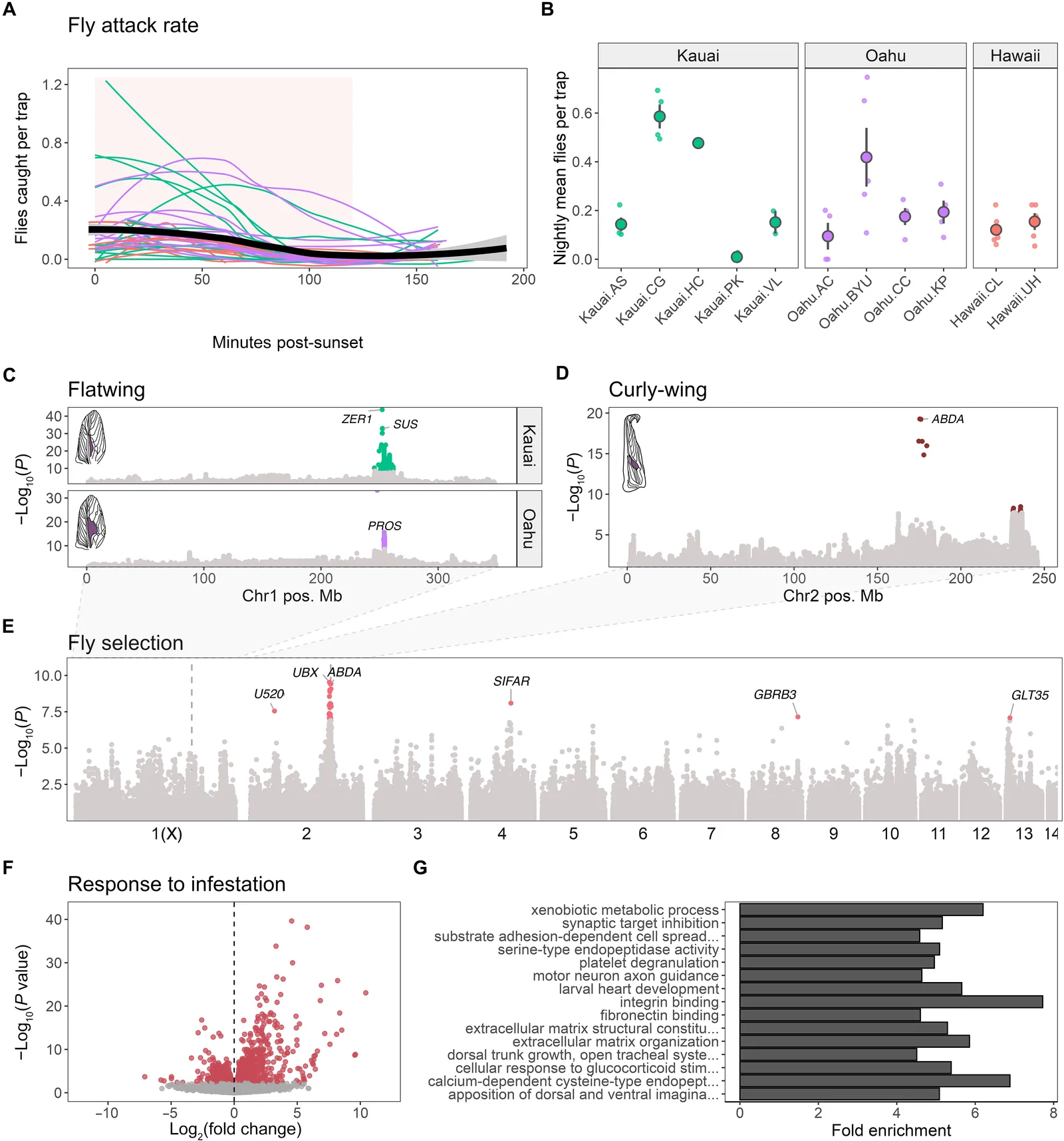
Variants associated with song-loss phenotypes and selection strength. (**A**) Numbers of flies caught per 10-min playback over time. Each colored line shows loess regression from a single night’s trials, colored by island, with the black line representing the average number of flies caught across all nights ±95% confidence intervals. (**B**) Log_2_-transformed relative fitness of silent males at each site. Small transparent points show estimates per night. Larger points show averages across nights ±1 SD to visualize variation rather than imply statistical significance. Averages above/below the dashed line at *y* = 0 indicate that silent males tend to have greater/lower fitness, respectively. (**C** and **D**) Variants and the statistical strength of their association with flatwing and curly-wing phenotypes. Significant (*P*_adj._ < 0.05) Fw-associated variants are highlighted in green and purple for Kauai and Oahu flatwing phenotypes, respectively, and Cw-associated variants are in dark red. Illustrations of flatwing and curly-wing phenotypes are adapted from (*13*). (**E**) Variants and the strength of their association with fly attack rates, with genes near variants with *P*_adj._ < 0.05 (red) highlighted. Flatwing and curly-wing-associated regions are indicated by dashed vertical lines. (**F**) Differential gene expression between body tissues of crickets that were uninfested or 4 days postinfestation with fly larvae. Significantly differentially expressed genes are highlighted in red. (**G**) Top 15 overrepresented gene ontology categories among differentially expressed genes.

Fly attack rates were highest in the first hours following sunset, after which they steadily declined (Fig. 4A), consistent with observations from North American mainland populations (*41*). Nightly attack rates were positively associated with increased precipitation (linear regression: *F*_1,29_ = 12.370, *P* = 0.001) but showed no relationship with temperature (*F*_1,29_ = 0.627, *P* = 0.400) and differed significantly between locations (location nested within island: *F*_8,29_ = 9.826, *P* < 0.001; full model *R*^2^ = 0.807). The nightly average likelihood of cricket song playback attracting one or more flies (averaged across the 2 hours following sunset) was significantly repeatable within locations (linear mixed model with log-transformed response: *R* = 0.575, 95% confidence interval = [0.178, 0.801], *P* < 0.001). At one location with a high proportion of singing male crickets (Kauai.PK, Fig. 1), song playback attracted just a single fly in 36 trials across three nights. Conversely, song playback attracted non-negligible numbers of flies at all three locations in which singing male cricket phenotypes are absent, consistent with previous, anecdotal observations (*42*) (Fig. 4B).

We used average fly attack rates at each site to infer the relative fitness of singing-capable and mutant reduced-song males in each location, informed by prior knowledge and further simplifying assumptions regarding typical life span, the likelihood of infestation having fatal consequences, and the time taken for larvae to develop and kill the host. These are fully described in Materials and Methods and are only approximations, given uncertainty of measures from prior studies, which include measures from related field cricket species. We estimate relative fitness of reduced-song males, specifically in the context of fly parasitism, to be high but greatly variable across sites, with mean 2.082 ± 0.841 SD. However, life span is just one component of fitness, and silent males are disadvantaged in the context of attracting female mates. A previous study that compared the frequency of singing males in field surveys with that of the offspring of wild-caught females estimated the relative fitness of silent males in the context of reduced mate attraction at 0.609 (*22*). Assuming the reduced fitness of silent males is reasonably consistent across locations, we thus estimate the net relative fitness of silent males across both contexts at 1.268 ± 0.512 SD, indicating a variable direction of selection across locations (minimum relative fitness = 0.609 at Kauai.PK, maximum = 2.257 at Kauai.CG) and, in many cases, across nights (fig. S9).

### Selection influences the frequency of a recently evolved male-silencing morph

Testing whether genomic regions associated with male-silencing wing morphs, flatwing and curly-wing, show evidence of selection related to fly attack rates requires knowledge of the region(s) underpinning each morph’s expression. Each morph has previously been subjected to genome-wide association studies but with modest sample sizes (or reduced representation sequencing) and earlier genome assemblies for *T. oceanicus* (*10*, *12*, *19*, *21*), so we reperformed these analyses. A complication is that at least two and perhaps three convergent flatwing phenotypes appear to have arisen independently via independent mutations in the region of the gene encoding doublesex (*10*, *12*) on Chr1 (the X chromosome); genetic knockout and knockdown of doublesex is associated with flatwing-like phenotypes in other cricket species (*43*). Prior studies have indicated that these genetic associations are stratified by island (*10*, *12*), so we identified variants that are consistently flatwing-associated on both Kauai and Oahu (with too few flatwing phenotypes surveyed from Hawaii for a similar analysis), among which it is reasonable to expect signatures of selection across islands. This analysis highlighted 10 Fw-associated variants shared across islands (*P*_adj._ < 0.05 in Kauai and Oahu), all of which were annotated for the nearest gene *prospero*, adjacent to the gene encoding doublesex, at 254 Mb on Chr1 (Fig. 4C). Curly-wing-associated loci were located on Chr2, an autosome, with the strength of association peaking in the region of two hox genes at 176 Mb (Fig. 4D): *ABDA* and *UBX*.

We compared genetic variation across the 300 cricket genomes from all 11 locations with the nightly average number of flies attracted per cricket song playback (i.e., selection from the fly), using a latent factor mixed model (LFMM) (*44*). QQ plots of observed versus expected *P* values indicated a strong overrepresentation of low *P* values in the empirical data (fig. S10), representing evidence of contemporary local adaptation. Of 40 significantly (*P*_adj._ < 0.05) selection-associated variants identified in this analysis, 33 (82.5%) were within 5 Mb of the consistently curly-wing-associated locus at 176 Mb on Chr2 and were annotated for proximity to curly-wing-associated genes *ABDA* and *UBX* (Fig. 4E). The flatwing-associated region was not strongly highlighted, perhaps influenced by its multiple genetic architectures, which could reduce statistical power to detect an association, or because it spread less recently than curly-wing and has fixed in some locations (*8*, *17*). We also did not observe significant signals of selection across broader chromosomal regions, as might be expected if selection from flies influenced the frequency of SVs, although a minimum of two of the six 10-Mb windows containing one or more significantly selected variants were within the region of polymorphic SVs (fig. S11).

Besides those in the region of the Cw-associated locus, other selected variants were within or nearby four more genes distributed across the rest of the genome—annotated for similarity with *Drosophila* genes *SIFAR*, *U520*, *GBRB3*, and *GLT35*—which might reflect selection on other traits important in evading or suppressing larval infestation. Notably, *GLT35* influences tracheal development, with mutations in this gene leading to malformed tracheal tubes in *Drosophila* (*45*). Tracheal development is directly implicated in cricket responses to larval infestation due to the apparent ability of *O. ochracea* larvae to co-opt hosts’ immune encapsulation responses to construct trachea that they use to avoid suffocating (*46*). To explore further, we interrogated publicly available RNA sequencing data from body tissue of Hawaiian *T. oceanicus* that were uninfested or had been infested for 4 days (*47*). We found 1099 genes (of 13,374 in the analysis) significantly differed in expression after 4 days of infestation (Fig. 4F). None were highlighted by our selection analysis above; however, differentially expressed (*P*_adj._ < 0.05) genes were overrepresented in gene ontology categories “serine-type endopeptidase activity” and “integrin binding,” both associated with immune encapsulation (*48*, *49*), and for genes involved in “dorsal trunk growth, open tracheal system” (all *P* ≤ 3.10 × 10^−5^) (Fig. 4G). The overrepresentation of these genes supports the view that encapsulation and tracheal development play important roles in larval infestation and present a potential target for selection.

## DISCUSSION

Understanding the conditions that promote genetic adaptation is a primary goal in evolutionary biology. This has attained pressing importance, given the need to understand whether and how wild populations will adapt to selection pressures increasingly imposed on them by human activity. The recent introduction of a parasitoid fly to Hawaii exposed local *T. oceanicus* populations to a novel and extreme selection pressure, and our study offers rare insights into the circumstances during and following rapid genetic adaptation. The strength of selection imposed by the fly is evidenced by severe recent declines in effective population size and by field assays of fly attack rate, with the latter revealing that contemporary selection remains strong at most sites despite the spread of male-silencing phenotypes. Differences in contemporary selection strength are associated with the frequency of alleles in the region most strongly associated with the male-silencing “curly-wing” phenotype, which recently emerged and spread in multiple locations (*11*, *20*), and other genes corresponding to traits of interest in the coevolutionary interaction between crickets and the eavesdropping parasitoid fly.

Much research in evolutionary biology and ecology concerns the ability of wild populations to adapt to selection, but the process of genetic adaptation is costly, particularly in small populations. Rapid genetic adaptation to strong selection pressures necessarily entails genetic bottlenecks, eroding the genetic variation on which future selection can act and potentially increasing the risk of exposure to harmful mutations (*50*). In Hawaiian *T. oceanicus*, multiple lines of inquiry support marked declines in effective population sizes following the human introduction of *O. ochracea*. These populations now face not only ongoing selection from the fly—which, as discussed below, remains present at every site we sampled—but also elevated risk of negative consequences of genetic drift and reduced ability to respond to further environmental perturbations. Besides imposing genetic bottlenecks, anthropogenic selection pressures can also negatively affect populations in the longer term by drawing them away from previous local fitness optima, potentially creating the conditions for evolutionary traps (*51*). Early behavioral studies indicated the fly’s introduction to Hawaii fostered the spread of male-silencing cricket morphs (*8*), which are now observed at moderate to high frequency at every location we surveyed but have not been recorded elsewhere in the species’ range (*13*). Yet, despite its disadvantages in attracting flies and other predators, male cricket song is an important sexual signal, with extensive experiments revealing silent males are strongly disadvantaged in the context of sexual attraction (*19**,* *20**,* *22**,* *37*). Moreover, male song has important social consequences, with males and females each investing more in reproductive tissues when reared in the presence of conspecific male song (*38**,* *39*). It is therefore plausible that the spread of male-silencing phenotypes could have negatively affected the persistence and resilience of cricket populations by reducing reproductive output.

Our estimates of relative fitness suggest that silent males are strongly favored at three of the 11 locations surveyed due to high fly attack rates but are selected against in one population, Kauai.PK, due to the relatively low risk of singing males attracting parasitoid flies and countervailing fitness costs in the context of sexual selection. Across the remainder, the direction of selection was inferred to vary across nights. Unexpectedly, these include three locations at which singing-capable males have been absent for several years (*11*, *52*), which would be expected to greatly reduce parasitoid fly populations (*53*), as predicted following the initial emergence of the flatwing phenotype (*8*). That we nevertheless observe non-negligible fly attack rates at these sites, even after fixation of male-silencing mutations, is curious. However, a recent study showed flies are also attracted to the song of other Grylline crickets that co-occur with *T. oceanicus* in Hawaii, but which are infrequently observed (e.g., *Gryllus bimaculatus*) or offer less viable hosts for larval development (e.g., *G. sigillatus*) (*42*). This raises the intriguing prospect that *O. ochracea* remain in the vicinity of silent *T. oceanicus* populations by expressing phonotaxis toward the acoustic signals of other co-occurring cricket species, complicating the coevolutionary interaction. An alternative possibility is that flies are itinerant or were attracted to song playbacks at the site of silent populations from nearby locations. We have not heard singing males in recent surveys of areas surrounding these sites, but the likely migration asymmetry between flies and crickets represents an important consideration for future research as this is crucial to predictions of local adaptation and coevolution in host-parasite interactions (*54*). A caveat to our estimates of the relative fitness of singing and silent males is that these rely on estimates of typical life span and infestation success rate from other cricket species and do not account for potential pleiotropic effects of male-silencing mutations (*21*, *55*) or differences in population density (*17**,* *36*) as we lack comprehensive data on these factors.

Besides male-silencing phenotypes, we detected statistical associations between contemporary selection and allele frequencies within or near genes corresponding to traits that attracted interest in early behavioral studies investigating adaptive responses to the parasitoid fly (*56*–*58*)—in some cases, before the emergence of silent male morphs. One such variant is within the gene *SIFAR*, which in *Drosophila* influences variation in wing extension index during male courtship (*59*). Prior studies in Hawaiian *T. oceanicus* explored whether singing behavior differs across populations (*56*, *60*, *61*), with an early study reporting evidence that singing-capable males in parasitized populations reduced calling effort during the interval of peak fly activity (*56*). More recently, the prediction that selection could act to modulate singing behavior has been largely sidelined following the spread of adaptive male-silencing phenotypes. We also observed evidence of selection on the gene *GLT35*, which influences tracheal development, with mutations in this gene leading to malformed tracheal tubes in *Drosophila* (*45*). This, too, relates to prior research in this system. As in other insect hosts of parasitoids, crickets mount an immune encapsulation response against infesting *O. ochracea* larvae (*57*). However, *O. ochracea* larvae appear to co-opt this encapsulation response to construct a tracheal funnel which attaches to the cricket’s abdominal wall, allowing developing larvae to avoid suffocation (*46*). Our analysis of differences in gene expression between uninfested and infested cricket body tissues (*47*) showed infestation-responsive genes are overrepresented not only for functions involved in larval encapsulation but also tracheal development. The apparent trade-off between host encapsulation response and larval co-option has led to speculation that selection might act on attenuated encapsulation responses (*57*), but it could also feasibly act on genes, such as *GLT35*, that influence tracheal development. Whether evidence of selection on genes implicated in singing behavior and tracheal development reflects adaptive variation in these traits represents an interesting avenue for further research, particularly if variants affecting these traits might be favored due to circumventing negative fitness consequences of male song loss in the context of sexual selection.

Inversion polymorphisms are a topic of enduring interest in evolutionary biology (*62*), but the sources of balancing selection involved in their maintenance, as well as their consequences for evolutionary responses to selection, are still not well understood (*46*). We have recently shown that genomic regions underlying flatwing and curly-wing phenotypes are statistically associated with, but are not caused by, large segregating inversions (*19*) and unexpectedly found evidence of many further large segregating SVs, which we suspect to be chromosomal inversions. Most of these SVs were polymorphic in *T. oceanicus* populations across all three Hawaiian islands, indicating their likely maintenance under balancing selection, an interpretation reinforced by evidence several also persist in Australian populations in the core species range. Balancing selection could act to retain genetic variation in these regions for a number of reasons (*63*), including (associative) overdominance (*64**,* *65*), negative frequency-dependent selection, epistasis or antagonistic pleiotropy (*66*), and sexual antagonism (*67*). Whatever the contribution of these different sources of selection, SV polymorphisms account for a very substantial portion of the genetic variation in Hawaiian cricket populations and have pronounced effects on gene expression, hinting at important phenotypic and fitness effects (*28*). Balanced polymorphisms are expected to contribute disproportionally to adaptive potential by contributing to “functional” genetic diversity (*68*), i.e., that with important effects on phenotype and fitness (*69*). How this trades off with negative consequences of suppressed recombination between alternative karyotypes maintained by balancing selection and selective inference more generally (*70*) in influencing adaptive potential is an open question and one of considerable importance. Given the strength of selection that is likely to be necessary to maintain inversion polymorphisms in several replicate small population following severe genetic bottlenecks and a recent history of rapid genetic adaptation, Hawaiian *T. oceanicus* appears to offer a compelling system to comprehensively test the sources of balancing selection maintaining, and the adaptive importance of, structural polymorphisms.

## MATERIALS AND METHODS

### Selection assays

We performed 489 fly capture trials starting at sunset on 42 nights at 11 patches, in January to February and June to July in 2022. Each fly capture trial involved eight traps, half of which broadcasted a cricket song model based on average song parameters across several Hawaiian populations of *T. oceanicus* (*71*), and lasted 10 min, with song broadcast at 85 dB at a distance of 5 cm using a speaker (Jam HX-P303BK) attached to an MP3 player (SanDisk SDMX26-008G-E46P). Control traps were identical to those broadcasting song but without playback. Traps were set out in a 3 × 3 square, with 1.5 m between adjacent traps and an alternating order of control and song traps, which were swapped each trial. Trials began at sunset, with 12 trials per night (unless interrupted by heavy rain). After trials, captured flies were released 10 m from the trap, after which we waited for 3 min before resuming trials. We recorded the number of flies attracted to each trap. One site, Kauai.WC, was only assayed on a single night in October 2022 as we found it later than the other locations. Because fly abundance was repeatable across nights at other sites (see the main text), it did not appear to differ between surveys performed in early and mid-2022, and because assays at this site reported similar rates of fly attack as the nearest site, Kauai.CG, we retained this site in our analysis.

Repeatability of fly attack rates across nights were estimated using rptR v0.9.22 (*72*) by running linear mixed models with a response variable of the log-adjusted fly attack rate within 2 hours of sunset, averaged across nights. Data for average daily temperature and precipitation at each location were retrieved using the nasapower R package.

### Estimating expected life span

Attracting a single fly has drastic fitness costs, as infestation by *O. ochracea* larvae is very often fatal and gravid females are remarkably accurate in locating the source of cricket song (*15*, *16*) even after the cessation of singing behavior (*73*). To estimate the expected adult life span of singing-capable males at different locations, from which to infer the strength of selection against song, we made the following assumptions based on existing data. Assumption 1: We estimated the typical adult life span in the wild of reduced-song (e.g., expressing curly-wing or flatwing morphs) males at 29 days, based on longevity records from a wild population of a related field cricket, *Gryllus campestris* (average longevity of 28.9 days ± 0.53 SE) (*74*) and from *T. oceanicus*’ sister species, *T. commodus*, reared under seminatural conditions (29.07 days ± 1.67 SE) (*75*). Assumption 2: Males sing for an average of 7.6 min per night in the two hours following sunset, as observed in a prior study (*61*), and begin singing shortly after adult eclosion [singing behavior was recorded at 0 to 3 days posteclosion by (*76*)]. Assumption 3: Phonotaxis by gravid female *O. ochracea* has fatal consequences for *T. oceanicus* males 61% of the time, based on findings from a related cricket host, *Gryllus texensis*, in which deposited planidia successfully established in, emerged from and killed 61% of males (*77*). Last, assumption 4: Cessation of reproductive viability occurs at 6 days postinfestation; Adamo *et al.* (*78*) found reproductive viability strongly declines after 5 days of infestation, with larvae emerging and killing crickets 7 to 10 days postinfestation.

Following the assumptions stated above, we calculated the number of flies a singing-capable male cricket attracts per day as *N*_fly_ = mean number of flies attracted per 10-min playback * 0.76 (assumption 2). Then, on the basis of assumption 3

$$P\left(\text{infestation}\right)=1-{\left(1-0.61\right)}^{N_{\text{fly}}}$$

where *P*(infestation) is the daily probability of becoming fatally infested. We further assume that the life span of a cricket follows a geometric distribution, with a time lag *L* between infection and loss of reproductive viability (6 days; see assumption 4 above). Thus, a singing male’s expected reproductive life span is

$$E\left(\text{reproductive life span}\right)=\frac{1}{P\left(\text{infestation}\right)}+L$$

On top of this, we imposed a maximum expected reproductive life span of 29 days, the same as that of silent males. From this, we estimated the average reproductive life span of singing-capable males at 16.514 days ± 7.054 SD across nights/locations.

### Sample collection, extraction, and sequencing

Tissue samples were collected from wild-caught males at 11 locations between July 2021 and June 2022 by removing a single hindleg. There are no mandatory permissions for collection of tissue samples from non-native, nonendangered insects in Hawaii. Samples from two populations (Oahu.AC and Oahu.BYU) were supplemented with samples collected in October 2018 (*N* = 10 of 30, *N* = 5 of 30, respectively). After tissue collection, animals were released, and crickets missing a hindleg were not sampled to avoid resampling. Legs were stored in 100% ethanol at −80°C until DNA extraction using a CTAB-chloroform protocol. Libraries were prepared using NEBNext Ultra II FS Kits with ∼300–base pair (bp) inserts, and sequenced at the University of Liverpool Centre for Genomic Research on an Illumina NovaSeq S4 flow cell, generating paired-end 150-bp reads. Ninety of these sequenced samples were used in a previously published analysis (*19*), and we also included previously resequenced genomes from seven Australian *T. oceanicus* and three Australian *T. commodus* (a sister species) (*12*).

### Variant identification, calling, and filtering

Reads were provided by the facility after initial quality and adapter trimming using Cutadapt v1.2.1104 (*79*) and Sickle v1.200, with a minimum window quality score of 20. Further trimming was performed using FastP v.0.24.0 with default parameters. Reads were aligned to the *T. oceanicus* genome v3 (ENA accession GCA_976985615) using BWA-mem v.0.7.19 with default parameters, following which polymerase chain reaction duplicates were flagged using SAMBAMBA v.1.0.1. We performed variant discovery from aligned reads of 55 samples, five per location, selecting samples with high sequencing depth and which represented all morphological variants recorded at the respective location. Variant discovery and calling of samples was performed in Freebayes v1.3.6 (*80*). Variants were initially filtered using vcftools v0.1.16 (*81*) to retain genotype calls with: sequencing depth greater than 5x and less than 60x; minimum genotype quality ≥ 20; mapping quality ≥ 30; minimum genotype call rate across samples ≥ 0.75; biallelic variants (*N* = 75,180,819 variants). Variants with minor allele frequency < 0.05 (or minor allele frequency < 0.1 in genetic association and selection analyses) were also removed unless otherwise indicated. Weir and Cockerham’s *F*_ST_ was calculated in 50-kb windows with 10-kb step size in vcftools.

### Analyses of RAD sequencing data

We cleaned, aligned, and called variants from previously published RAD sequencing data from Australian *T. oceanicus* following the same methods described for our whole-genome sequencing data. We then filtered the variants for minor allele frequency ≥ 0.1, biallelic sites, minimum depth 5x, missingness of up to 0.9 and minimum genotype quality of 20. We merged the resulting variant call format (VCF) file with the full Hawaiian VCF using bcftools and used vcftools tools to filter the merged VCF for minor allele frequency ≥ 0.05, biallelic sites, and maximum missingness of 0.5. Windowed PCA was performed across 10-Mb regions with an increment of 100 kb, using winpca (*82*).

### Windowed PCA and linkage analysis

We used the Lostruct R package to run windowed PCA (*23*), often used to identify putative chromosomal inversions, across the genome. We first further filtered the VCF for single-nucleotide polymorphisms (SNPs) with less than 1% missing genotypes, samples missing genotypes at less than 20% of loci, and retained only one variant per 50 kb (*N* = 149,673; 294 samples). We converted the VCF to TPED format and implemented PCA in 50-SNP windows across the genome. To visualize linkage across chromosomes, we thinned the set of SNPs using a window of 200 kb, filtered for minor allele frequency > 0.2, and then calculated and plotted *r*^2^ values in LDheatmap (*83*).

### Population structure and demographic inference

Inferences of population structure and demographic history were performed using linkage-pruned (plink: indep-pairwise 50 10 0.1) variants genotyped in ≥90% of samples and located on chromosomes 5, 9, and 14 (those not showing evidence of massive SVs) (*N* = 73,674 variants). Phylogenetic relationships were explored using FastTree (*30*) and plotted using the R package ggtree (*84*). *F*-branch statistics were calculated in Dsuite (*32*).

Repetitive regions were excluded before running analyses to estimate long-term changes in effective population size, inferred using samples collected from Oahu.BYU (to which *T. oceanicus* were first introduced) in 2022. We ran this analysis using samples from Oahu.BYU as *T. oceanicus* appears to have been first introduced to Oahu and Oahu.BYU had the highest estimated *N*_e_ in recent inferences based on linkage disequilibrium (see below), but patterns were strongly consistent across populations on Oahu (fig. S7). This analysis was performed in Phlash (*34*), which implements a Bayesian method for inferring population size history from recombining genomic sequence data based on coalescent theory. Estimates were scaled by assuming a Drosophila-like mutation rate of 3.32 × 10^−9^ (*85*).

To explore more recent shifts in *N*_e_, we used GONE2, a method based on patterns of linkage disequilibrium (*35**,* *86*). This tool was run using the filtered vcf described above, further filtered to retain only genotypes present in ≥90% of samples, and without linkage pruning. A small number of samples collected before 2021 were removed, and Kauai.WC was not included in the analysis due to the smaller sample size (*N* = 17). We ran this analysis using 1 million SNPs across chromosomes 5, 9, and 14, assuming a recombination rate of 1.5 centimorgans (cM)/Mb based on estimates from other cricket species (*87*). We verified that adopting a rate of 1 cM/Mb had little impact on interpretation of recent declines, although this resulted in fluctuating *N*_e_ in the last few generations that could suggest poor model fit (*86*) (fig. S1). To assess reliability of results, we reran the analysis 20 times for each population, using random subsets of 20 samples and 100,000 SNPs.

### Genome-wide association and selection tests

Genome-wide association tests for curly-wing and flatwing phenotypes were implemented in GEMMA (*88*), using linear mixed models with a genetic relatedness matrix included as a random effect, and tested using likelihood ratio tests. Tests for loci that differed in frequency in association with the average nightly number of flies attracted to cricket song at each site were implemented using LFMMs that account for hidden population structure and are implemented in the R package LFMM (*44*). We used ridge penalty estimates and a *K* value of 3, which was determined from inspection of PCA scree plots and the number of islands. *P* values were calibrated by a genomic inflation factor. Input data were pruned for linkage disequilibrium in 50-variant windows using Plink (--indep-pairwise 50 10 0.1), variants missing in >90% of individuals or with minor allele frequency < 0.1 were removed, and missing genotypes were imputed using the impute function in the R package LEA (*89*).

### Gene expression analyses

Trimmed, paired-end RNA sequencing data collected from neural tissue of 48 lab-reared *T. oceanicus* of both sexes (*29*), and body tissues from 18 *T. oceanicus* that were or were not infected with *O. ochracea* larvae (*47*), were downloaded from the NCBI Sequence Read Archive. In each case, reads filtered with FastP and default parameters and then aligned to the *T. oceanicus* genome using HISAT2 with default parameters (*90*), after which gene counts were estimated using StringTie (*91*). In addition, for the 48 samples from (*29*), variant calling was performed using bcftools v.1.21, and filtered for genotype quality (>20), missingness (<0.25) and minor allele frequency (>0.05), before running chromosome-level PCA in Plink (*92*). Variance partitioning of gene expression counts was performed using the R package variancePartition v1.34.0 (*93*), and differential expression analyses were performed in DESeq2 v1.44.0 (*94*). Gene ontology overrepresentation tests for biological processes were performed using topGO v2.56.0 (*95*).
