# Supplementary material for: Genomic signatures of adaptation across a landscape of crickets following the introduction of a parasitoid fly
Source: Sci Adv. 2026 Sep 9;12(37):eaed1906. doi: 10.1126/sciadv.aed1906 (PMC13557055; doi:10.1126/sciadv.aed1906)
Supplement: Supplementary file 1 [file sciadv.aed1906_sm.pdf]

Science Advances
AAAS

Supplementary Materials for

Genomic signatures of adaptation across a landscape of crickets following the introduction of a parasitoid fly

Jack G. Rayner *et al.*

Corresponding author: Nathan W. Bailey, nwb3@st-andrews.ac.uk

Sci. Adv. **12**, eaed1906 (2026)
DOI: 10.1126/sciadv.aed1906

This PDF file includes:

Figs. S1 to S12

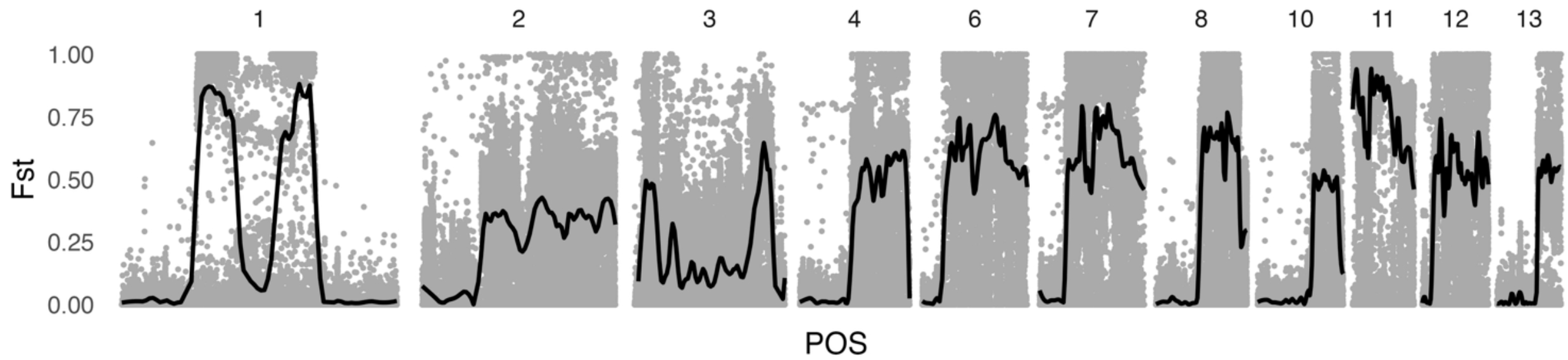

Fig. S1. Genomic divergence associated with SVs.

F_{ST} values, shown on the Y-Axis between alternative homokaryotypes based on whole-chromosome PC1 values, are elevated in the region of structural variants. F_{ST} values were calculated using just cricket samples from Kauai to avoid confounding effects of genetic divergence between island populations. This does not account for all of the large SVs we observed (e.g., we observed two large SVs on Chr2), but only those that contributed most strongly to variance on PC1. Lines illustrate LOESS trended means.

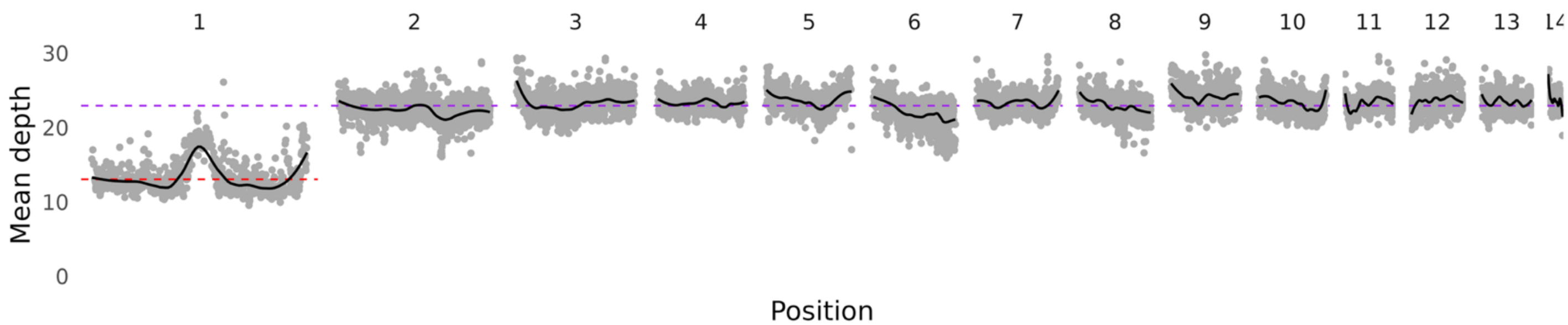

Fig. S2. Mean sequencing depth along each chromosome. Black lines show trends from LOESS regression. Purple lines show average read depth across filtered autosomal variants. The red line shows average read depth on Chr1, the X chromosome.

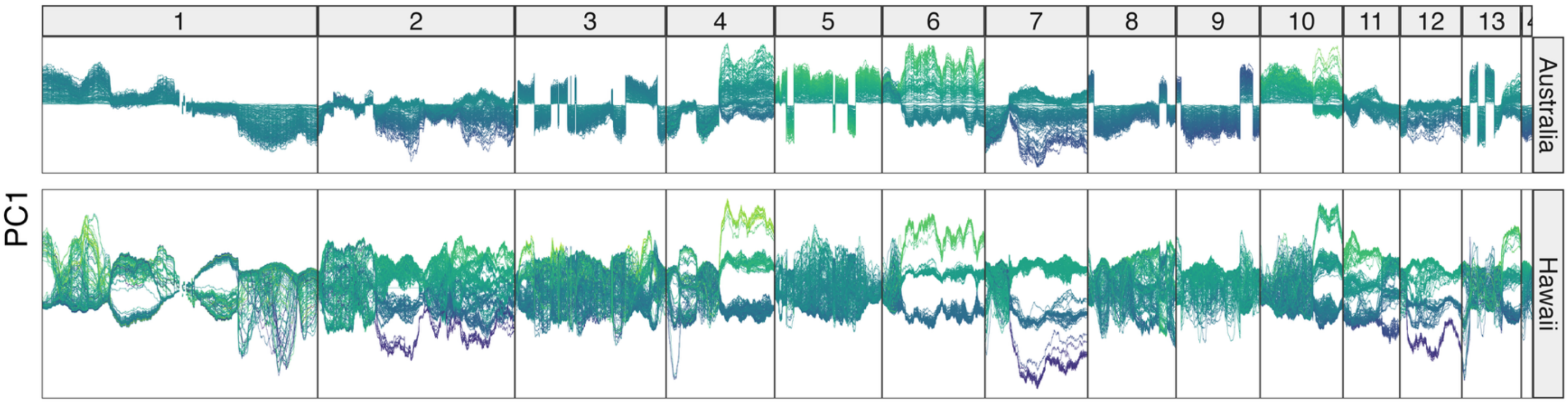

Fig S3. Comparison of genomic divergence between geographic regions.
The first principal component from windowed PCA is shown, illustrating patterns of genomic divergence between individuals (lines) across a subset of chromosomes in Australian (top panel) and Hawaiian (bottom panel) *T. oceanicus*. Lines are coloured by mean PC1 score per chromosome.

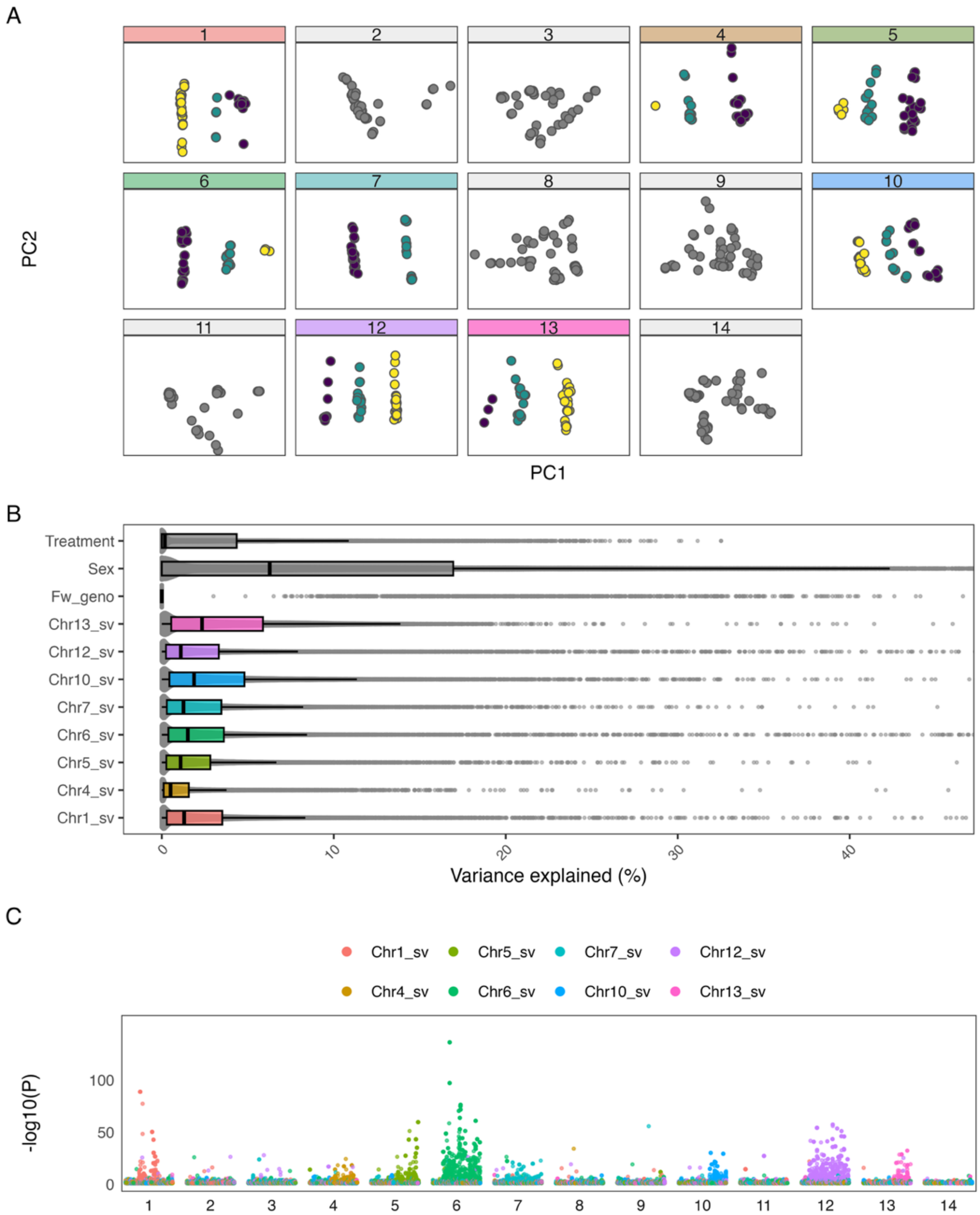

Fig. S4. Effects of SVs on gene expression. A) PCA of filtered variants from RNA-seq data separated by chromosome, with discrete clustering of samples indicative of one or more large structural variants. Samples are coloured based on assigned karyotype based on PC1 values. **B)** Distributions of the proportion of variation in gene expression across 25,359 genes that is accounted for by structural variants on different chromosomes (coloured), and other variables included in the original analysis by (*26*). *Treatment* refers to the presence of male cricket song playback during rearing (Y/N), and *Fw_geno* refers to genotype at the *flatwing* locus. **C)** The genomic distribution of genes included in the analysis, with greater values on the Y-axis indicative of stronger evidence of differential expression. Points are coloured according to the structural variant included as a predictor variable in the differential expression analysis. For example, the cluster of strongly differentially expressed green points on chromosome 6 indicate that the structural variant identified on this chromosome had strong effects on gene expression.

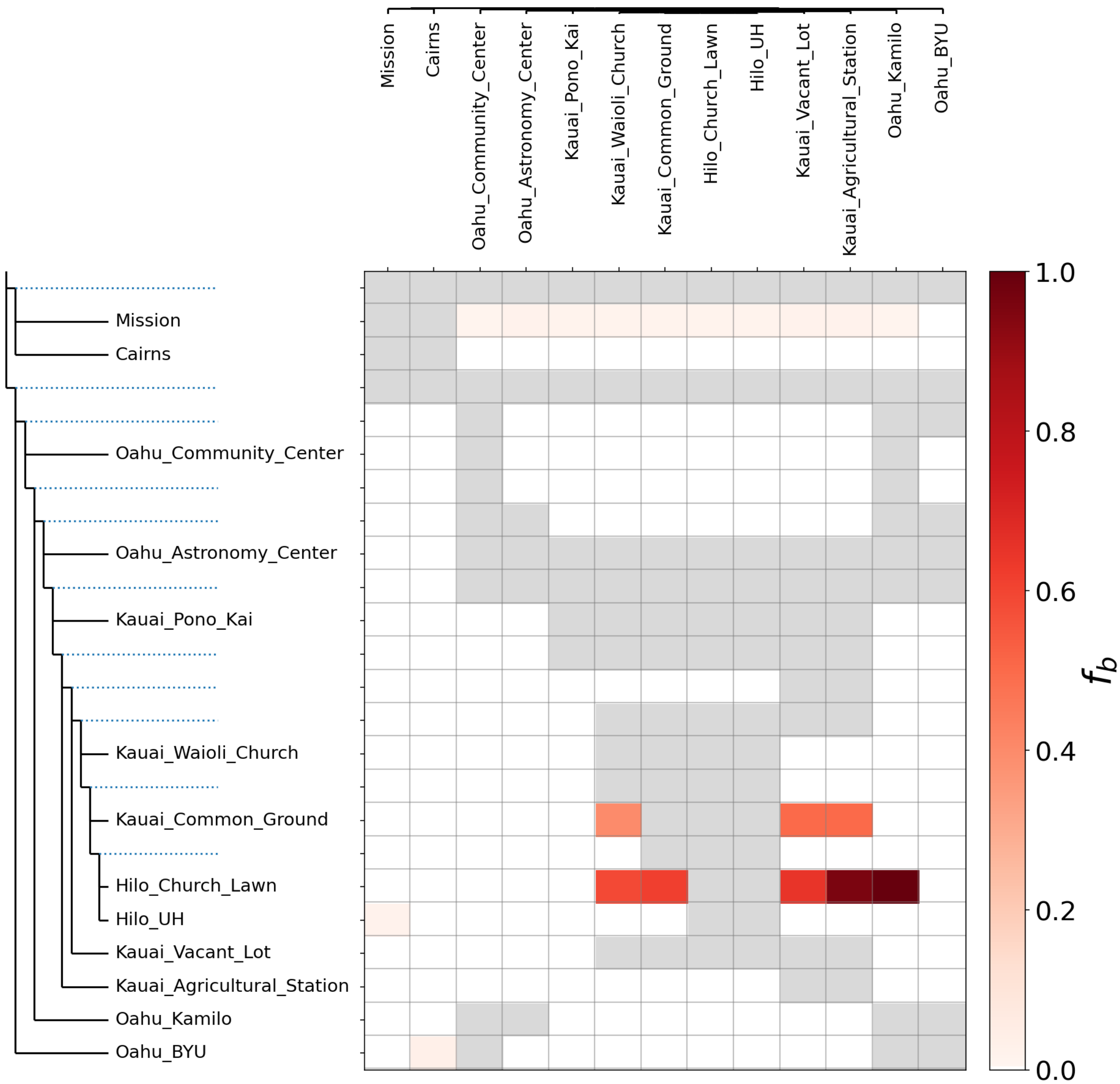

Fig. S5. Evidence of gene flow.
F-branch statistics showing evidence of excess allele-sharing between multiple populations, indicative of gene flow.

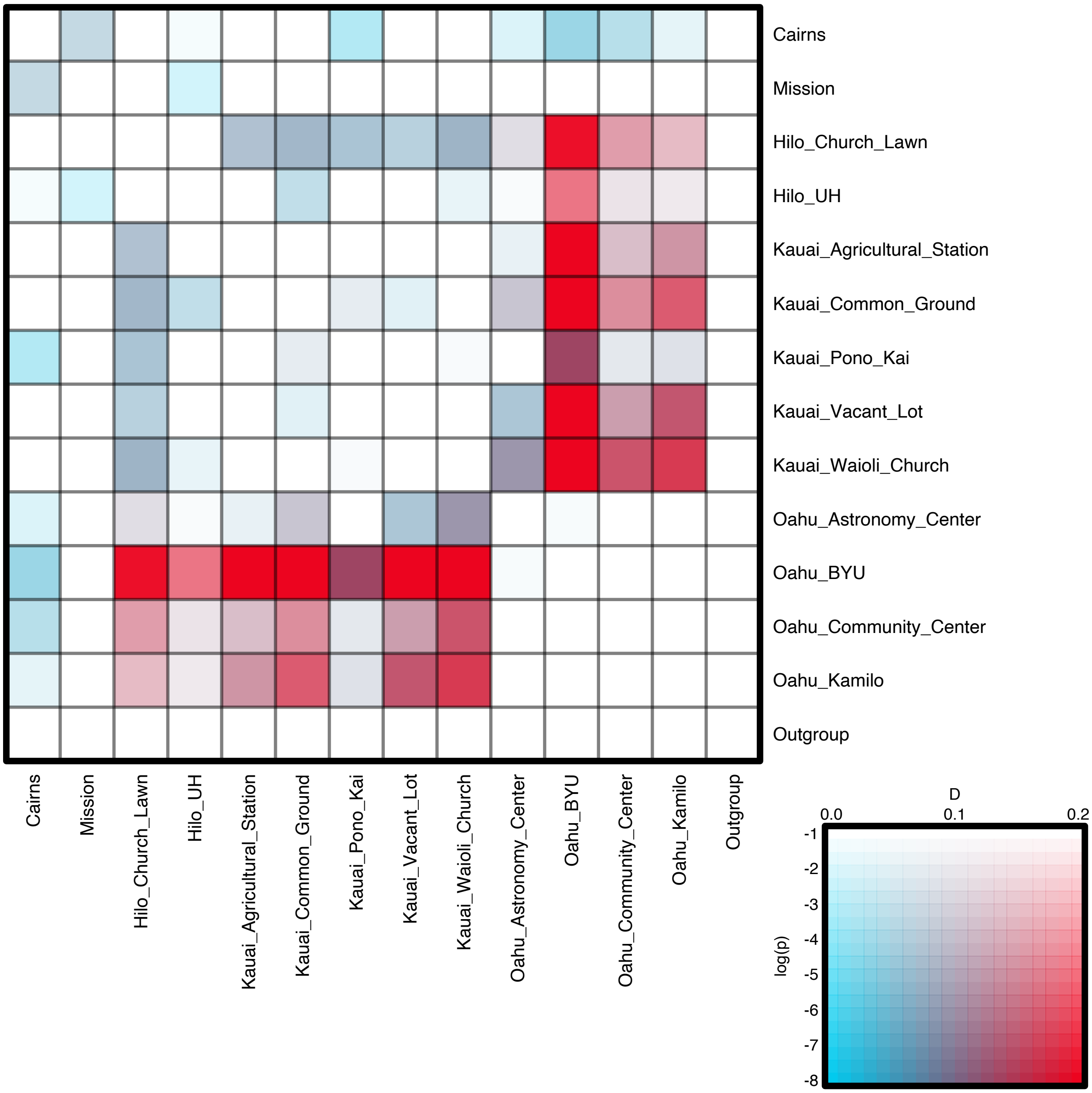

Fig. S6. Excess allele sharing between populations.
ABBA-BABA statistics summarised across population pairs, coloured by D (hue) and P-value (saturation).

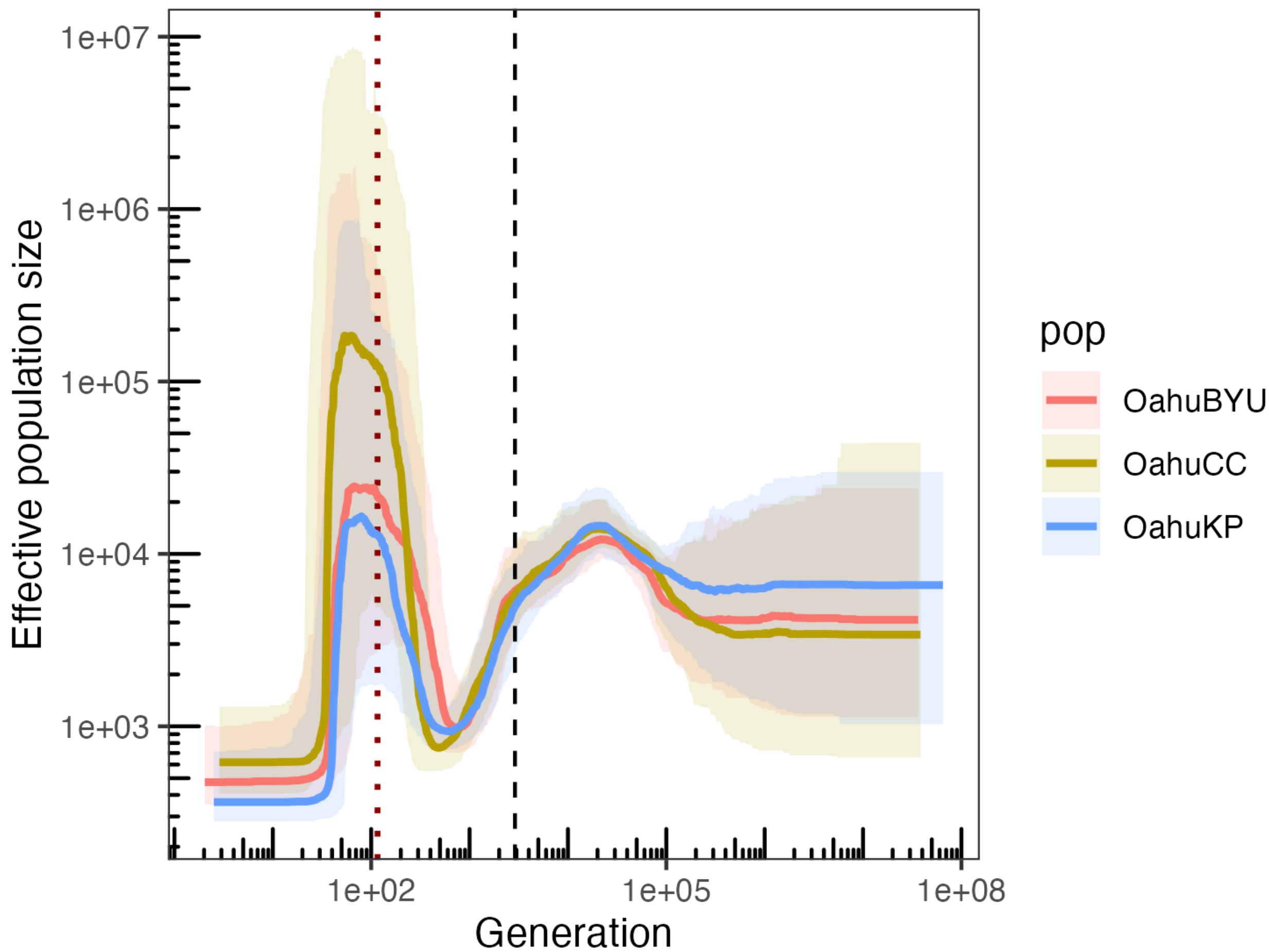

Fig. S7. Long-term changes in N_e.
Data shown from multiple Oahu population.Lines represent estimates of the fly's introduction (dotted, red) and the split of Hawaiian *T. oceanicus* from Australian core populations (dashed, black). Ribbons illustrate 95% posterior confidence intervals.

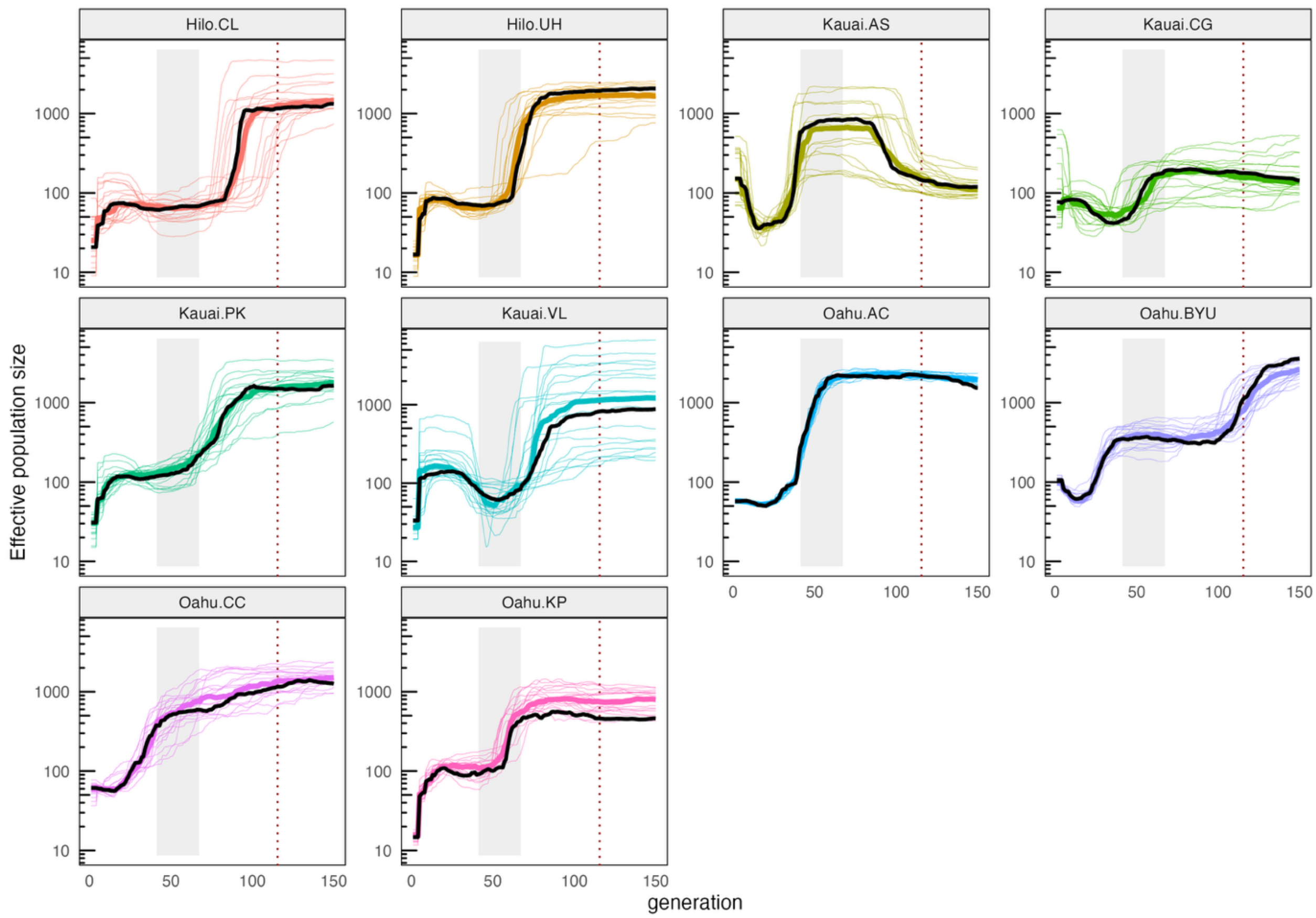

Fig. S8. Short-term changes in N_e in random subsamples from 10 populations.
Each thin coloured line shows inferences from a random subset of 20 samples and 100,000 SNPs. The thicker coloured line indicates the median average across all analyses, and the black lines illustrate the trend from the full analysis of each population, shown in Fig. 3. The dotted vertical line indicates the first observation of the parasitoid fly in the Hawaiian archipelago, and the grey rectangle indicates the interval in which the first male-silencing variants were observed to emerge and spread.

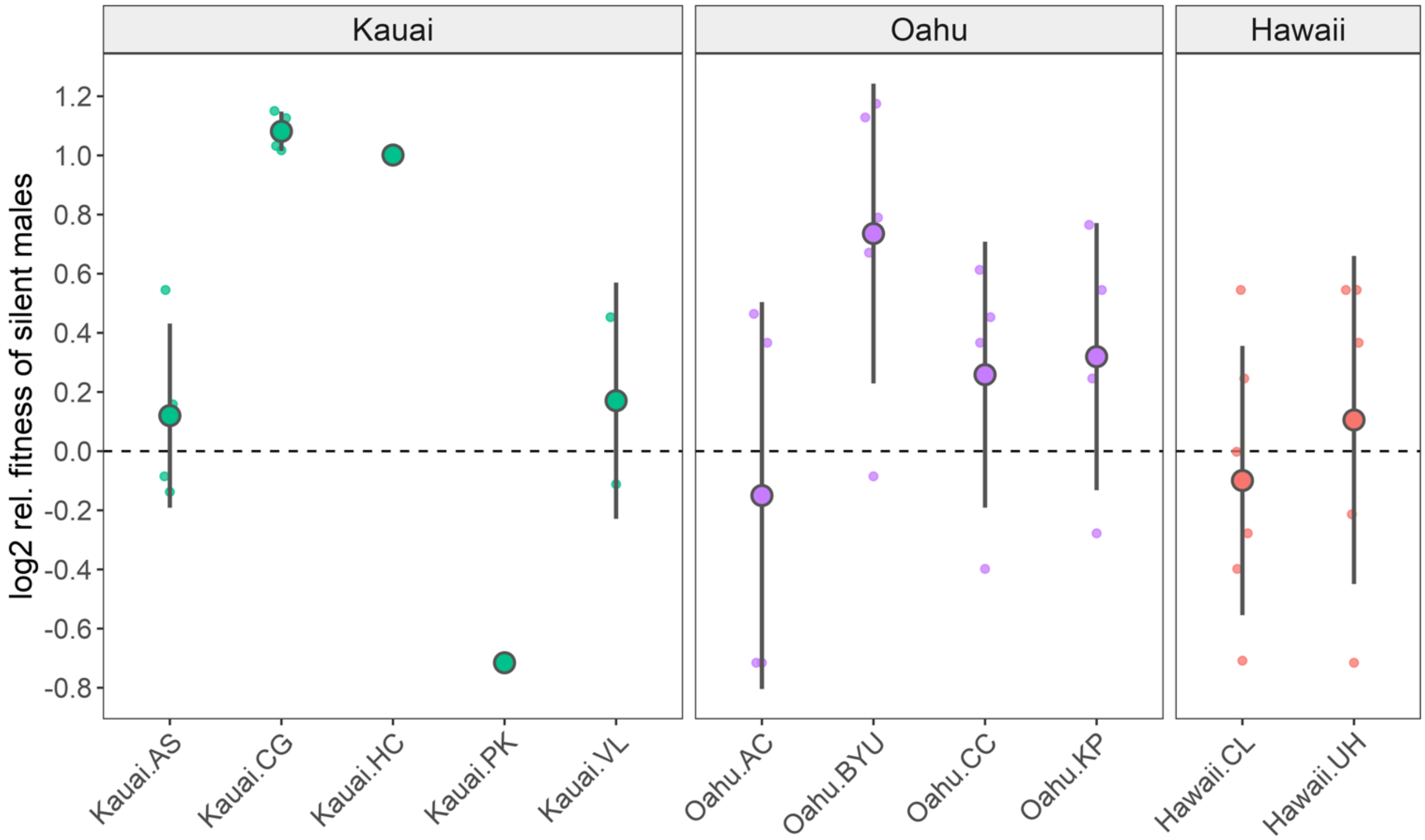

Fig. S9. Relative fitness inferences.
Log2-transformed relative fitness of silent males at each site based on estimates described in the main text. Small transparent points show estimates per night. Larger points show averages across nights ± 1 standard deviation, to visualise variation rather than imply statistical significance. Averages above/below the dashed line at y=0 indicate that silent males tend to have greater/lower fitness, respectively.

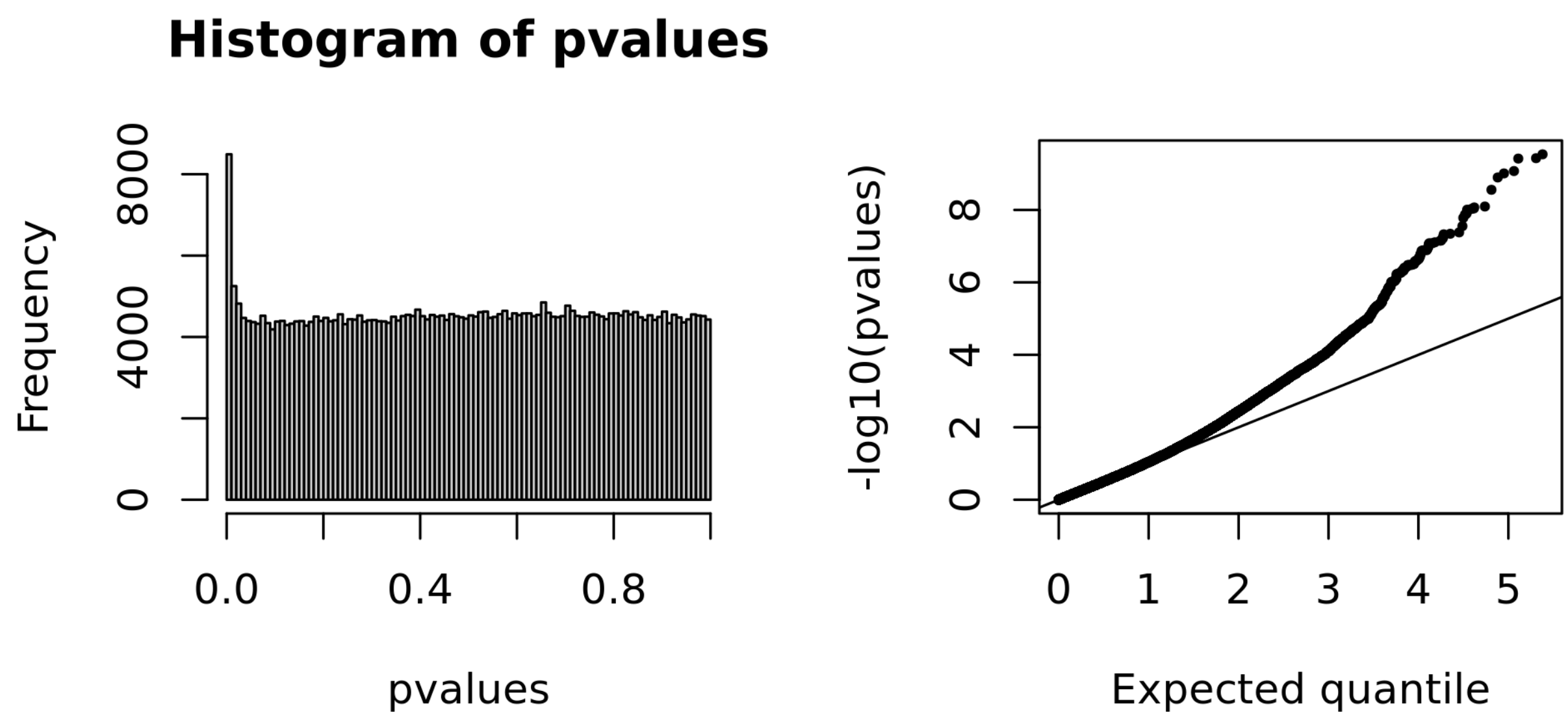

Fig. S10. Evidence of local adaptation.
A histogram of calibrated P-values from the LFMM used to test evidence of fly selection on cricket genome variants, and a qqplot illustrating the inflation of low P-values relative to expectation.

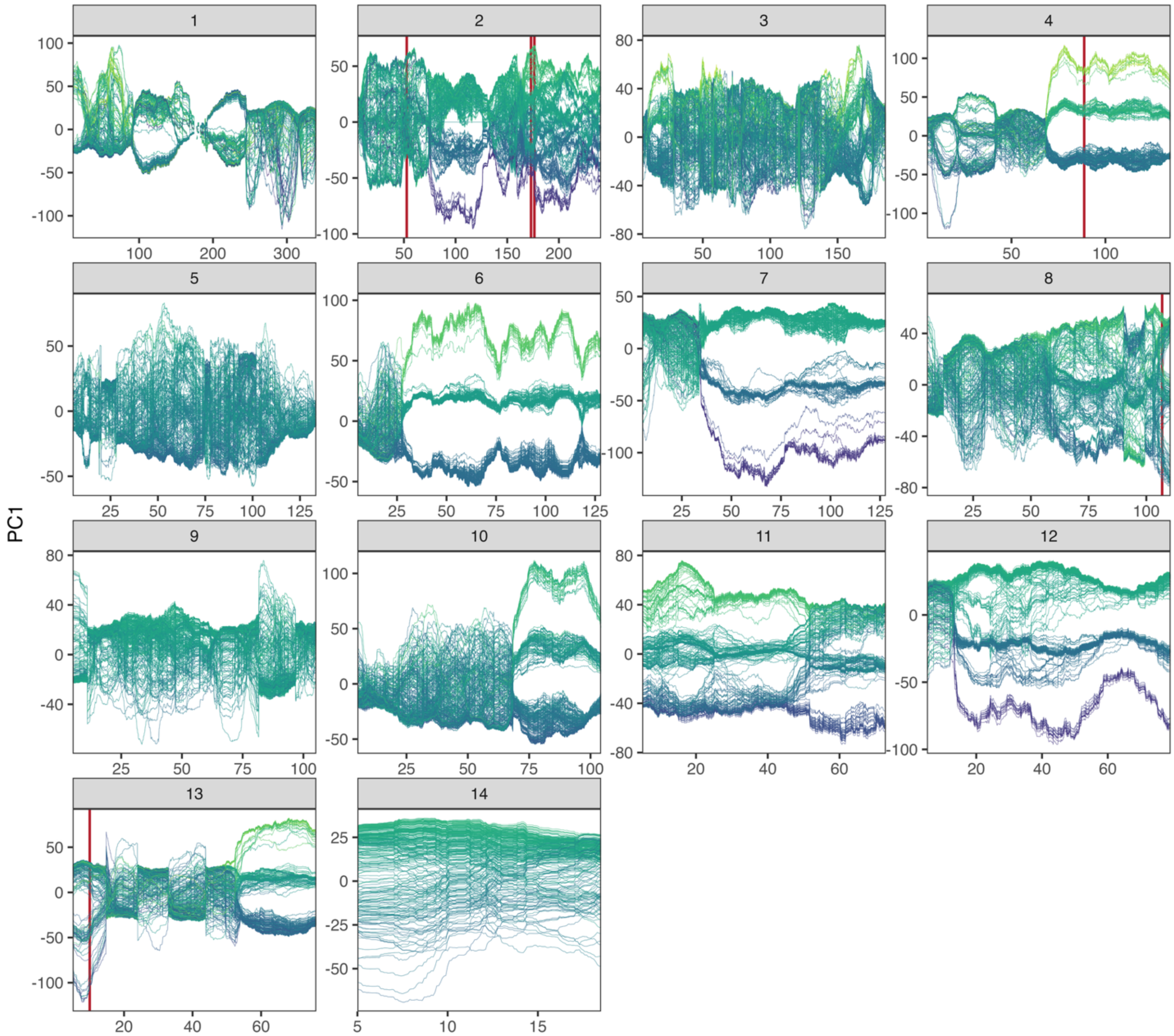

Fig S11. **Locations of selected variants with respect to SVs**.
Windowed PCA plots (see Fig 1C) with the positions of alleles that are significantly associated with the strength of selection from the fly highlighted by red vertical lines.

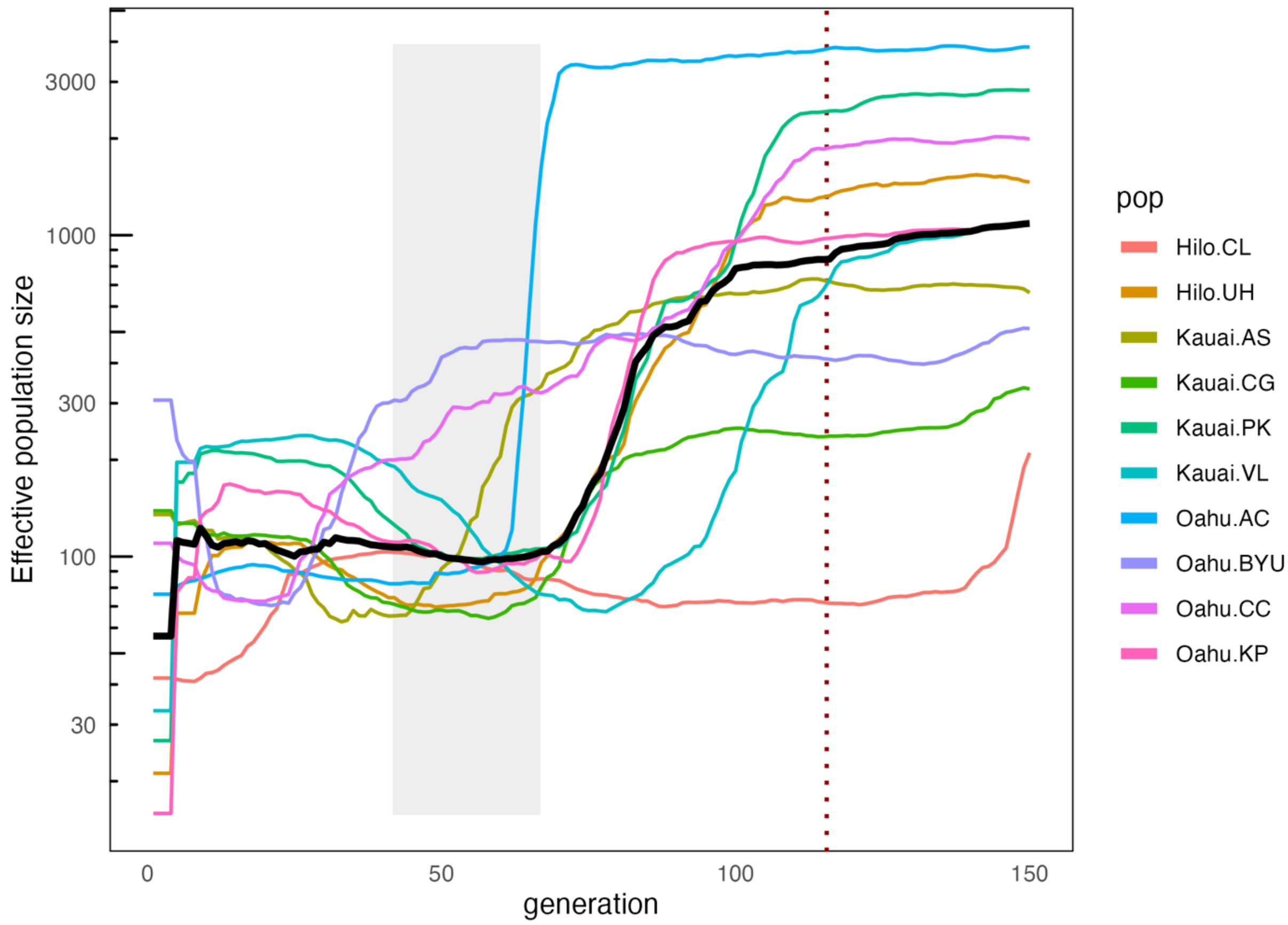

Fig. S12. Short-term changes in N_e assuming a recombination rate of 1Cm/Mb. This differs from analyses based on an assumed recombination rate of 1.5Cm/Mb reported in Fig. 3E.
